# 2-(3*H*-1,3-Benzothia­zol-2-yl­idene)­propane­dial

**DOI:** 10.1107/S1600536811030248

**Published:** 2011-08-06

**Authors:** Hamid Ennajih, Rachid Bouhfid, Stephane Massip, Jean Michel Leger, El Mokhtar Essassi

**Affiliations:** a Moroccan Advanced Science, Innovation and Research (MASCIR) Foundation – INANOTECH, ENSET, Avenue de l’Armée Royale, Madinat El Irfane 10100, Rabat, Morocco; b Laboratoire de Chimie Physique et Minérale, EA4138 Pharmacochimie, Université Victor Ségalen Bordeaux 2, 146 Rue Léo Saignat, 33076 Bordeaux Cedex, France; c Laboratoire de Chimie Organique Hétérocyclique, Faculté des Sciences, Avenue Ibn Battouta, BP 1014, Rabat, Morocco

## Abstract

In the title compound, C_10_H_7_NO_2_S, the dihedral angle between the benzimidazole and malonaldehyde group is 1.41 (2)°. An intra­molecular hydrogen bond is formed between the NH group and one of the adjacent carbonyl O atoms. In addition, the NH group forms an inter­molecular hydrogen bond to a symmetry equivalent of this carbonyl O atom, connecting the mol­ecules into centrosymmetric dimers. The structure also contains C—H⋯O inter­molecular inter­actions.

## Related literature

For biological activities of benzothia­zole derivatives, see: Mortimer *et al.* (2006 ▶); Yoshida *et al.* (2005 ▶); Vicini *et al.* (2003 ▶).
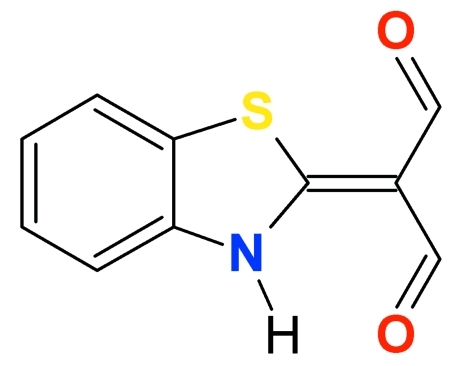


## Experimental

### 

#### Crystal data

C_10_H_7_NO_2_S
*M* *_r_* = 205.23
Monoclinic, 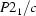

*a* = 8.3927 (10) Å
*b* = 5.0972 (8) Å
*c* = 20.739 (2) Å
β = 100.098 (8)°
*V* = 873.4 (2) Å^3^
*Z* = 4
Cu *K*α radiation
μ = 3.05 mm^−1^
*T* = 133 K
0.12 × 0.12 × 0.02 mm

#### Data collection

Rigaku R-AXIS RAPID diffractometer
Absorption correction: multi-scan (*CrystalClear*; Rigaku/MSC, 2005 ▶) *T*_min_ = 0.711, *T*_max_ = 0.942
10660 measured reflections
1569 independent reflections
1475 reflections with *I* > 2σ(*I*)
*R*_int_ = 0.082

#### Refinement

*R*[*F* ^2^ > 2σ(*F* ^2^)] = 0.067
*wR*(*F* ^2^) = 0.165
*S* = 1.02
1569 reflections
128 parameters
H-atom parameters constrained
Δρ_max_ = 0.60 e Å^−3^
Δρ_min_ = −0.45 e Å^−3^

### 

Data collection: *CrystalClear* (Rigaku/MSC, 2005 ▶); cell refinement: *CrystalClear*; data reduction: *CrystalClear*; program(s) used to solve structure: *SHELXS97* (Sheldrick, 2008 ▶); program(s) used to refine structure: *SHELXL97* (Sheldrick, 2008 ▶); molecular graphics: *PLATON* (van der Sluis & Spek, 1990 ▶; Spek, 2009 ▶); software used to prepare material for publication: *publCIF* (Westrip, 2010 ▶).

## Supplementary Material

Crystal structure: contains datablock(s) I, global. DOI: 10.1107/S1600536811030248/hg5064sup1.cif

Structure factors: contains datablock(s) I. DOI: 10.1107/S1600536811030248/hg5064Isup2.hkl

Supplementary material file. DOI: 10.1107/S1600536811030248/hg5064Isup3.cml

Additional supplementary materials:  crystallographic information; 3D view; checkCIF report

## Figures and Tables

**Table 1:** Hydrogen-bond geometry (Å, °)

*D*—H⋯*A*	*D*—H	H⋯*A*	*D*⋯*A*	*D*—H⋯*A*
N6—H6⋯O11	0.88	2.13	2.731 (3)	125
N6—H6⋯O11^i^	0.88	2.13	2.926 (3)	151
C3—H3⋯O14^ii^	0.95	2.43	3.297 (4)	152

Symmetry codes: (i) 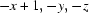; (ii) 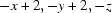.

**Fig. 1.**
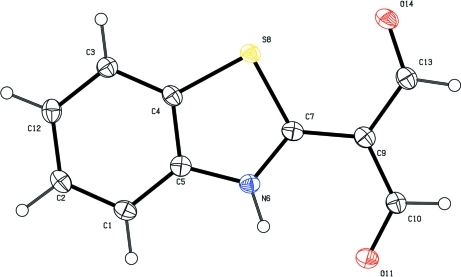
ORTEP diagram of the title molecule with the atom numbering scheme. Displacement ellipsoid are drawn at 30% probability level.

**Fig. 2.**
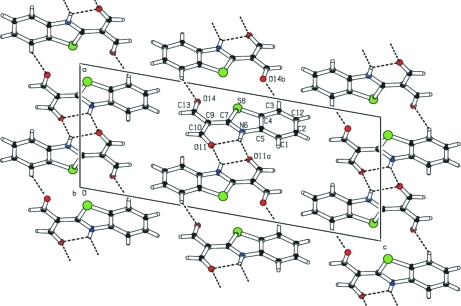
Packing diagram of the title compound viewed down the b axis. Dashed lines indicate hydrogen bonds intra and intermolecular interactions.

## supplementary crystallographic information

### Comment

Benzothiazole and its derivatives are good candidates that have fluorescent properties and possess diverse biological properties, such as antibacterial, anti-inflammatory and antitumor. (Mortimer *et al.* (2006); Yoshida *et al.* (2005); Vicini *et al.* (2003)). In the present paper, we report the synthesis of 1,3-benzothiazol-2(3*H*)-ylidenemalonaldehyde using the Vilsmeier reaction. This molecule can be used as a precursor for the synthesis of a variety of fluorescent molecules. The crystal structure of the title compound is characterized by bifurcated hydrogen bonds between the amine and aldehyde groups. The donor atom N6 gives the mean interactions with 2 acceptor atom O11. One is intramolecular (*x*, *y*, *z*) but the other one is intermolecular (1 - *x*, -*y*, -*z*). These interactions form a coplanar dimer around the centre in 1/2, 1/2, 0.5. Atom C3 in the molecule at (*x*, *y*, *z*) acts as a hydrogen-bond donor *via* atom H3 to atom O14 in the molecule at (2 - *x*, 2 - *y*, -*z*)

### Experimental

To *N*,*N*-dimethylformamide (2 ml) cooled in an ice bath was added dropwise phosphorus oxychloride (1.6 ml, 17.4 mmol) with stirring at below 5 °C. After this addition, a solution of 2-methybenzothiazole (2.9 mmol, 0.432 g) in DMF (2 ml) was added dropwise. The cooling bath was removed and the reaction mixture was stirred at 80 °C for 12 h. The resulting solution was added to ice-cooled water and made alkaline with NaOH (aq.) solution. The resulting precipitate was collected by filtration after 24 h, dried in air, recrystallized from ethanol, to give 1,3-benzothiazol-2(*3H*)-ylidenemalonaldehyde as colourless crystals.

### Refinement

Carbon and Nitrogen H-atoms were located from Fourier Fourier synthesis and placed in calculated positions (C—H 0.95 Å, N—H 0.88 Å) and included in the refinement in the riding model approximation with *U_iso_*(H) = 1.2*U_eq_*(C,N).

### Crystal data

**Table:**

C_10_H_7_NO_2_S	*F*(000) = 424
*M**_r_* = 205.23	*D*_x_ = 1.561 Mg m^−^^3^
Monoclinic, *P*2_1_/*c*	Cu *K*α radiation, λ = 1.54180 Å
Hall symbol: -P 2ybc	Cell parameters from 1475 reflections
*a* = 8.3927 (10) Å	θ = 7.5–71.9°
*b* = 5.0972 (8) Å	µ = 3.05 mm^−^^1^
*c* = 20.739 (2) Å	*T* = 133 K
β = 100.098 (8)°	Plate, colourless
*V* = 873.4 (2) Å^3^	0.12 × 0.12 × 0.02 mm
*Z* = 4	

### Data collection

**Table:**

Rigaku R-AXIS RAPID diffractometer	1569 independent reflections
Radiation source: micro-focus rotating anode	1475 reflections with *I* > 2σ(*I*)
confocal	*R*_int_ = 0.082
ω scans	θ_max_ = 71.9°, θ_min_ = 7.5°
Absorption correction: multi-scan (*CrystalClear*; Rigaku/MSC, 2005)	*h* = −10→10
*T*_min_ = 0.711, *T*_max_ = 0.942	*k* = −4→5
10660 measured reflections	*l* = −25→25

### Refinement

**Table:**

Refinement on *F*^2^	Primary atom site location: structure-invariant direct methods
Least-squares matrix: full	Secondary atom site location: difference Fourier map
*R*[*F*^2^ > 2σ(*F*^2^)] = 0.067	Hydrogen site location: inferred from neighbouring sites
*wR*(*F*^2^) = 0.165	H-atom parameters constrained
*S* = 1.02	*w* = 1/[σ^2^(*F*_o_^2^) + (0.098*P*)^2^ + 1.503*P*] where *P* = (*F*_o_^2^ + 2*F*_c_^2^)/3
1569 reflections	(Δ/σ)_max_ < 0.001
128 parameters	Δρ_max_ = 0.60 e Å^−^^3^
0 restraints	Δρ_min_ = −0.45 e Å^−^^3^

### Special details

**Table:**

Geometry. All e.s.d.'s (except the e.s.d. in the dihedral angle between two l.s. planes) are estimated using the full covariance matrix. The cell e.s.d.'s are taken into account individually in the estimation of e.s.d.'s in distances, angles and torsion angles; correlations between e.s.d.'s in cell parameters are only used when they are defined by crystal symmetry. An approximate (isotropic) treatment of cell e.s.d.'s is used for estimating e.s.d.'s involving l.s. planes.
Refinement. Refinement of *F*^2^ against ALL reflections. The weighted *R*-factor *wR* and goodness of fit *S* are based on *F*^2^, conventional *R*-factors *R* are based on *F*, with *F* set to zero for negative *F*^2^. The threshold expression of *F*^2^ > σ(*F*^2^) is used only for calculating *R*-factors(gt) *etc*. and is not relevant to the choice of reflections for refinement. *R*-factors based on *F*^2^ are statistically about twice as large as those based on *F*, and *R*- factors based on ALL data will be even larger.

### Fractional atomic coordinates and isotropic or equivalent isotropic displacement parameters (Å^2^)

**Table:**

	*x*	*y*	*z*	*U*_iso_*/*U*_eq_	
C1	0.6870 (3)	0.3667 (6)	0.16032 (14)	0.0291 (7)	
H1	0.6162	0.2210	0.1600	0.035*	
C2	0.7501 (3)	0.4952 (7)	0.21783 (13)	0.0311 (7)	
H2	0.7216	0.4366	0.2578	0.037*	
C3	0.8986 (3)	0.7993 (6)	0.16137 (14)	0.0272 (6)	
H3	0.9706	0.9434	0.1619	0.033*	
C4	0.8343 (3)	0.6732 (6)	0.10311 (13)	0.0256 (6)	
C5	0.7315 (3)	0.4596 (6)	0.10291 (13)	0.0255 (6)	
N6	0.6804 (3)	0.3641 (5)	0.03972 (11)	0.0248 (6)	
H6	0.6145	0.2294	0.0313	0.030*	
C7	0.7391 (3)	0.4926 (6)	−0.00706 (13)	0.0248 (6)	
S8	0.86490 (8)	0.74932 (13)	0.02434 (3)	0.0253 (3)	
C9	0.7044 (3)	0.4279 (6)	−0.07455 (13)	0.0267 (7)	
C10	0.6013 (3)	0.2122 (6)	−0.09676 (14)	0.0267 (7)	
H10	0.5856	0.1748	−0.1423	0.032*	
O11	0.5306 (2)	0.0694 (4)	−0.06329 (9)	0.0303 (5)	
C12	0.8543 (4)	0.7080 (6)	0.21876 (14)	0.0308 (7)	
H12	0.8955	0.7919	0.2592	0.037*	
C13	0.7714 (3)	0.5777 (6)	−0.12103 (14)	0.0303 (7)	
H13	0.7447	0.5248	−0.1655	0.036*	
O14	0.8605 (3)	0.7691 (4)	−0.10910 (11)	0.0343 (6)	

### Atomic displacement parameters (Å^2^)

**Table:**

	*U*^11^	*U*^22^	*U*^33^	*U*^12^	*U*^13^	*U*^23^
C1	0.0248 (13)	0.0302 (18)	0.0337 (14)	0.0004 (12)	0.0085 (11)	0.0039 (12)
C2	0.0296 (14)	0.037 (2)	0.0280 (14)	0.0031 (13)	0.0094 (11)	0.0059 (12)
C3	0.0231 (13)	0.0296 (17)	0.0300 (14)	0.0009 (12)	0.0079 (11)	−0.0012 (12)
C4	0.0205 (12)	0.0312 (17)	0.0266 (13)	0.0034 (11)	0.0087 (10)	0.0026 (12)
C5	0.0197 (12)	0.0278 (17)	0.0294 (14)	0.0042 (11)	0.0052 (10)	−0.0001 (11)
N6	0.0200 (11)	0.0268 (15)	0.0287 (11)	−0.0004 (10)	0.0071 (8)	−0.0004 (9)
C7	0.0176 (12)	0.0264 (17)	0.0318 (14)	0.0032 (11)	0.0084 (10)	0.0007 (11)
S8	0.0225 (4)	0.0277 (5)	0.0274 (5)	−0.0021 (2)	0.0090 (3)	0.0003 (2)
C9	0.0213 (13)	0.0307 (18)	0.0295 (14)	0.0040 (11)	0.0082 (10)	0.0010 (11)
C10	0.0218 (13)	0.0316 (18)	0.0277 (14)	0.0040 (11)	0.0070 (11)	0.0002 (11)
O11	0.0262 (10)	0.0315 (13)	0.0341 (10)	−0.0017 (8)	0.0083 (8)	0.0019 (9)
C12	0.0295 (15)	0.0367 (19)	0.0263 (14)	0.0045 (12)	0.0053 (11)	−0.0022 (12)
C13	0.0276 (14)	0.0352 (19)	0.0303 (14)	0.0030 (13)	0.0109 (11)	0.0003 (12)
O14	0.0333 (12)	0.0377 (15)	0.0353 (12)	−0.0053 (9)	0.0151 (9)	0.0016 (9)

### Geometric parameters (Å, °)

**Table:**

C1—C2	1.382 (4)	N6—C7	1.334 (4)
C1—C5	1.392 (4)	N6—H6	0.8800
C1—H1	0.9500	C7—C9	1.418 (4)
C2—C12	1.391 (4)	C7—S8	1.735 (3)
C2—H2	0.9500	C9—C13	1.421 (4)
C3—C12	1.388 (4)	C9—C10	1.425 (4)
C3—C4	1.391 (4)	C10—O11	1.228 (4)
C3—H3	0.9500	C10—H10	0.9500
C4—C5	1.388 (4)	C12—H12	0.9500
C4—S8	1.741 (3)	C13—O14	1.228 (4)
C5—N6	1.393 (3)	C13—H13	0.9500
			
C2—C1—C5	117.2 (3)	C5—N6—H6	122.6
C2—C1—H1	121.4	N6—C7—C9	124.4 (3)
C5—C1—H1	121.4	N6—C7—S8	112.0 (2)
C1—C2—C12	121.8 (3)	C9—C7—S8	123.5 (2)
C1—C2—H2	119.1	C7—S8—C4	90.23 (13)
C12—C2—H2	119.1	C7—C9—C13	120.5 (3)
C12—C3—C4	117.9 (3)	C7—C9—C10	120.4 (3)
C12—C3—H3	121.0	C13—C9—C10	119.1 (3)
C4—C3—H3	121.0	O11—C10—C9	126.9 (3)
C5—C4—C3	120.7 (3)	O11—C10—H10	116.5
C5—C4—S8	111.4 (2)	C9—C10—H10	116.5
C3—C4—S8	127.8 (2)	C3—C12—C2	120.8 (3)
C4—C5—C1	121.6 (3)	C3—C12—H12	119.6
C4—C5—N6	111.4 (2)	C2—C12—H12	119.6
C1—C5—N6	126.9 (3)	O14—C13—C9	126.2 (3)
C7—N6—C5	114.9 (2)	O14—C13—H13	116.9
C7—N6—H6	122.6	C9—C13—H13	116.9
			
C5—C1—C2—C12	−0.2 (4)	C9—C7—S8—C4	−179.5 (2)
C12—C3—C4—C5	−1.2 (4)	C5—C4—S8—C7	0.0 (2)
C12—C3—C4—S8	178.5 (2)	C3—C4—S8—C7	−179.7 (3)
C3—C4—C5—C1	1.1 (4)	N6—C7—C9—C13	179.4 (2)
S8—C4—C5—C1	−178.7 (2)	S8—C7—C9—C13	−1.0 (4)
C3—C4—C5—N6	179.7 (3)	N6—C7—C9—C10	−0.3 (4)
S8—C4—C5—N6	−0.1 (3)	S8—C7—C9—C10	179.3 (2)
C2—C1—C5—C4	−0.3 (4)	C7—C9—C10—O11	2.1 (4)
C2—C1—C5—N6	−178.7 (3)	C13—C9—C10—O11	−177.7 (3)
C4—C5—N6—C7	0.2 (3)	C4—C3—C12—C2	0.7 (4)
C1—C5—N6—C7	178.7 (3)	C1—C2—C12—C3	0.0 (5)
C5—N6—C7—C9	179.4 (2)	C7—C9—C13—O14	−0.4 (5)
C5—N6—C7—S8	−0.2 (3)	C10—C9—C13—O14	179.3 (3)
N6—C7—S8—C4	0.1 (2)		

### Hydrogen-bond geometry (Å, °)

**Table:**

*D*—H···*A*	*D*—H	H···*A*	*D*···*A*	*D*—H···*A*
N6—H6···O11	0.88	2.13	2.731 (3)	125.
N6—H6···O11^i^	0.88	2.13	2.926 (3)	151.
C3—H3···O14^ii^	0.95	2.43	3.297 (4)	152.

Symmetry codes: (i) −*x*+1, −*y*, −*z*; (ii) −*x*+2, −*y*+2, −*z*.
